# Effects of Au Addition on the Performance of Thermal Electronic Noses Based on Porous Cu_2_O–SnO_2_ Nanospheres

**DOI:** 10.3390/nano14242052

**Published:** 2024-12-22

**Authors:** Matteo Tonezzer, Taro Ueda, Soichiro Torai, Koki Fujita, Yasuhiro Shimizu, Takeo Hyodo

**Affiliations:** 1 Department of Chemical and Geological Sciences, University of Cagliari, 09042 Monserrato, Italy; 2 Graduate School of Integrated Science and Technology, Nagasaki University, 1–14 Bunkyo-machi, Nagasaki 852-8521, Japan; taroueda@nagasaki-u.ac.jp; 3 Graduate School of Engineering, Nagasaki University, 1–14 Bunkyomachi, Nagasaki 852-8521, Japan; torai-souichirou@fujielectric.com (S.T.); bb52123633@ms.nagasaki-u.ac.jp (K.F.); shimizu@nagasaki-u.ac.jp (Y.S.)

**Keywords:** semiconductor gas sensor, tin oxide, copper oxide, gold, nanocomposite, thermal electronic nose, VOCs

## Abstract

The electronic nose is an increasingly useful tool in many fields and applications. Our thermal electronic nose approach, based on nanostructured metal oxide chemiresistors in a thermal gradient, has the advantage of being tiny and therefore integrable in portable and wearable devices. Obviously, a wise choice of the nanomaterial is crucial for the device’s performance and should therefore be carefully considered. Here we show how the addition of different amounts of Au (between 1 and 5 wt%) on Cu_2_O–SnO_2_ nanospheres affects the thermal electronic nose performance. Interestingly, the best performance is not achieved with the material offering the highest intrinsic selectivity. This confirms the importance of specific studies, since the performance of chemoresistive gas sensors does not linearly affect the performance of the electronic nose. By optimizing the amount of Au, the device achieved a perfect classification of the tested gases (acetone, ethanol, and toluene) and a good concentration estimation (with a mean absolute percentage error around 16%). These performances, combined with potentially smaller dimensions of less than 0.5 mm^2^, make this thermal electronic nose an ideal candidate for numerous applications, such as in the agri-food, environmental, and biomedical sectors.

## 1. Introduction

Growing urbanization and awareness of how air quality affects human health make gas sensors increasingly important in the environmental field [1,2]. Furthermore, these devices are transversal and can be used in many fields, for example to non-invasively assess the freshness of food products [3] or for early medical screening through breath analysis [4].

Semiconductor gas sensors (usually based on metal oxides) are among the most studied in the world due to their advantages, such as simplicity of fabrication and use and the resulting low cost [5,6]. Such a gas sensor transforms the chemical reactions that occur on its surface into an increase/decrease of charge carriers, i.e., into an easily processable electrical signal. The resistance of the metal oxide increases or decreases depending on the presence of oxidizing or reducing gases that capture or release electrons, which contribute to the electrical signal [7].

The latest generation of devices, based on nanostructured materials, takes advantage of the large specific surface area and excellent gas diffusivity within the material, which improve the sensing properties [8,9]. Unfortunately, the selectivity of chemoresistive gas sensors is limited, and therefore the nanomaterial must be thoroughly investigated and tuned to the specific desired application, depending on the gases to be detected and possible interferents. For example, to discriminate between patients who may have diabetes from healthy people (for rapid pre-screenings that reduce health care costs), it is necessary to accurately detect very low concentrations (hundreds of ppb) of acetone in the presence of other VOCs like ethanol and toluene [10,11]. To tune the properties of sensing materials, the most common and successful methods in recent years have been the coupling of a second metal oxide to the nanomaterial [12,13] or the addition of noble metals on their surface [14,15,16]. As demonstrated by Barsan’s group with in operando experiments, the interface between two p- and n-type metal oxides leads to the formation of p-n junctions, increases the number of chemical reactions and leads to improved performance and higher selectivity [12]. Surface functionalization with noble metal nanoparticles exploits their catalytic activity [17] to increase the response and decrease the working temperature [18]. Obviously, the functionalization must be targeted towards the application, since different metals have different effects on the performance [19], and the effect of gold nanoparticles on different metal oxides is different [20]. Recently, two metals are also used simultaneously [21]. Using both of these methods synergistically amplifies their effects [22,23].

We first synthesized porous tin oxide (pr-SnO_2_) spheres by ultrasonic spray pyrolysis using polymethyl methacrylate (PMMA) microspheres as a template in order to increase the specific surface area and the sensor response [24]. We then added Cu_2_O so that the interface between the two oxides affects the carrier density in order to improve the device performance [25], Finally, we added different amounts of Au to study the catalytic activity of VOC oxidation (acetone, ethanol, and toluene) and the influence on the sensor performance [26]. In this way we found that the maximum selectivity (2.34, acetone over ethanol) is obtained with the sample containing 3.0% Au.

Using an array of sensors in a so-called electronic nose mimics the mammalian nose (where signals from many different receptors are processed together by the brain) and allows for greatly improved selectivity [27]. A traditional electronic nose is composed of sensors made of different materials, in order to maximize the variance between their responses and the information fed to the machine learning algorithms that act as the brain of the system [28].

In this work, we use different nanomaterials (porous Cu_2_O–SnO_2_ spheres with different amounts of Au added) to simulate thermal electronic noses [29], i.e., we combine the device response at different temperatures. This innovative type of electronic nose uses a single material that works at different temperatures, instead of different materials. In this way, the entire device can be realized in less than 0.25 mm^2^ with standard deposition and lithography techniques used in microelectronics [30].

The aim of this study is to understand which nanomaterial (porous Cu_2_O–SnO_2_ nanospheres with different amounts of Au) is the best for the realization of a thermal electronic nose for the detection, discrimination, and quantification of acetone, ethanol, and toluene. A further aim is to possibly understand whether the intrinsic selectivity of the material used as a sensor is reflected in the performance of the electronic nose.

## 2. Materials and Methods

### 2.1. Synthesis of Porous Au–Cu_2_O–SnO_2_ Nanospheres

Porous Cu_2_O–SnO_2_ nanospheres were synthesized by ultrasonic spray pyrolysis, as previously reported in detail [24,25,26]. An amount of 40 cm^3^ of aqueous dispersion containing home-made PMMA microspheres (average size 70 nm) [31] was mixed with 60 cm^3^ of an aqueous SnCl_4_ solution (0.05 mol dm^−3^) containing appropriate amounts of CuCl_2_ and HAuCl_4_ [24]. The resulting solutions were ultrasonicated to mists and then introduced into an electric furnace at 1100 °C under an air flow of 1500 sccm to evaporate the water.

The resulting products are SnO_2_ powders containing Cu_2_O and an amount of Au that was adjusted from 1.0 to 5.0 wt% (Figure S1). The obtained powders were labeled from pr1 to pr5, where the number represents the wt% of Au in the porous Cu_2_O–SnO_2_ nanospheres.

### 2.2. Sensors Fabrication and Gas Detection Tests

Each powder was mixed with α-terpineol to form a paste that was then screen-printed onto a pair of interdigitated Pt electrodes spaced approximately 200 μm apart on an alumina substrate (Figure S2), and the devices were dried at 100 °C. The sensors were then calcined at 550 °C for 5 h in ambient air. The dynamic resistance of the sensors was measured using a DAQ 970A data acquisition system (Keysight Technologies, Santa Rosa, CA, USA).

The sensors were subjected to different concentrations (2.5, 5, 10, 20, 50, and 100 ppm) of three different VOCs (acetone, ethanol, and toluene), at temperatures between 300 and 500 °C in steps of 50 °C. Each concentration was set by mixing the target gas with dry air, maintaining the total flow at 100 sccm. The sensor response was defined as Ra/Rg, where Ra is the sensor resistance in air and Rg is the resistance in the presence of the VOC. The selectivity between two gases was defined as Resp(A)/Resp(B), where Resp(A) is the response to gas A and Resp(B) is the response to gas B. The overall selectivity of the sensor is the selectivity between the target gas (acetone) and the interferent with the highest response [32].

### 2.3. Thermal Electronic Nose

This innovative approach, developed by us on the basis of the pioneering work of Sysoev [33], replaces the use of different materials (including metal oxides, conductive polymers, graphene, carbon nanotubes, and 2D materials) with a simple thermal gradient. Since the response of a thermal oxide changes a lot with the working temperature, due to the many reactions that occur on its surface, the same material working at different temperatures can be as effective as sensors composed of different materials [34]. Moreover, this approach allows us to test the performances simply by simulating the thermal electronic nose, as in this case: testing the sensor at different temperatures and combining their responses before building the real prototype. The sensor response was calculated at each working temperature, obtaining a “thermal fingerprint”, then transformed into a point in the 5-dimensional space [35]. Principal component analysis (PCA) was used to reduce the dimensionality of the 5-dimensional space and visualize how the points from the different gases relate to each other [36]. Linear discriminant analysis (LDA) was used to maximize inter-class variance and minimize intra-class variance, in order to distinguish classes (in this case gases) [37,38]. A support vector machine (SVM) was used as a classifier to distinguish individual VOCs [39,40]. A linear kernel was used to ensure that the limited data did not result in overfitting [41]. These algorithms were used to evaluate how well the electronic nose can distinguish different gases [42,43]. Finally, a regression in the 5-dimensional space through an SVM was used to estimate the concentration of each gas. The mean absolute percentage error (MAPE) was used as a metric to evaluate the performance of each sensor.

## 3. Results and Discussion

**Figure 1:**
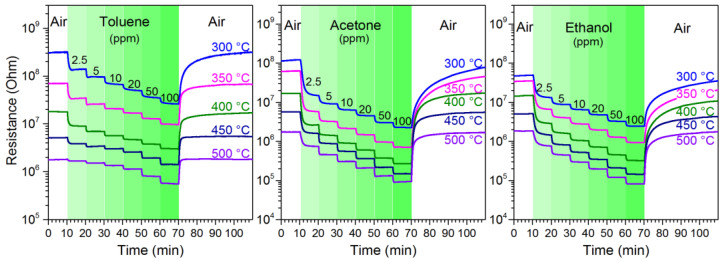
Dynamic resistance of the pr3 sensor as a function of temperature and concentration of toluene, acetone and ethanol.

The dynamic resistance of the Cu_2_O–SnO_2_ based sensors was measured at various concentrations and temperatures for different VOCs. Figure 1 shows, as an example, the dynamic resistance obtained with the sample pr3, i.e., with 3% Au. As expected, the resistance in air of the sensor decreases with increasing working temperature. Furthermore, the gas injection resistance decreases more and more as the concentration increases from 2.5 to 100 ppm.

**Figure 2:**
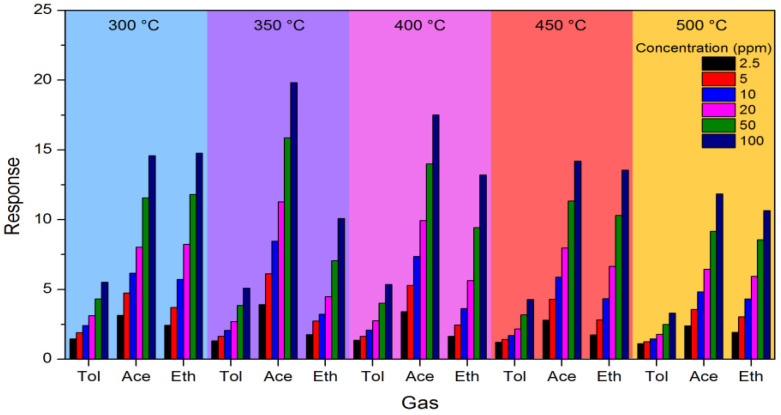
Response of sensor pr1 to various concentrations of the three VOCs at different working temperatures.

The sensor response was calculated for each concentration of each VOC at all five temperatures. Figure 2 shows an example of the calculated responses for sensor pr1.

Although the figure provides a lot of information, it is difficult to spot clear patterns. Toluene always gives a lower response than the other gases, while the ratio between the response to acetone and ethanol varies with both temperature and gas concentration. This is particularly evident for selectivity: for example, the selectivity between acetone and ethanol at 450 °C is 1.6 at 2.5 ppm (shortest black bars), while it decreases to 1.05 at 100 ppm (longest dark blue bars). The one clear and constant trend is that the response always increases with increasing gas concentration. It is therefore evident that averaging is needed to obtain an idea of the overall trend and compare the sensors and the working conditions.

**Figure 3:**
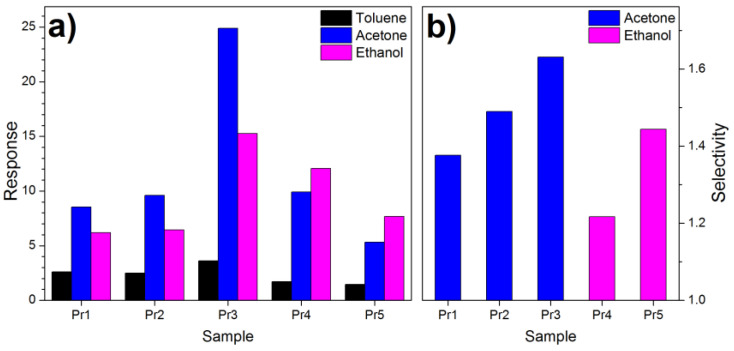
(**a**) Response and (**b**) Selectivity of samples with various amounts of Au. The color in (**b**) is related to the gas to which the sensor is most sensitive.

To calculate the intrinsic selectivity of the various materials used as sensors, the response of each material was averaged over all concentrations and at all temperatures. Figure 3a shows the average responses obtained, where it is clear that toluene always gives the lowest response.

Instead, the response to acetone and ethanol is not univocal: the nanocomposites with 1, 2, 3 wt% of Au are more sensitive to acetone, while those with 4, 5 wt% of Au are more sensitive to ethanol. The most selective material is the Cu_2_O–SnO_2_ with 3 wt% of Au (pr3) which has a selectivity of 1.63 for acetone compared to ethanol.

The results in Figure 3b seem to indicate that pr3 is the best material to fabricate an acetone sensor or an electronic nose, but the situation is more complicated. Figure S3 shows the selectivity of the various nanomaterials as a function of the working temperature and the gas concentration. The numbers in the figure express the ratio between the response to acetone and that to ethanol. If the number is greater than 1, it is the selectivity; otherwise, it is the reciprocal of the selectivity, since the material is, in that case, more sensitive to ethanol.

On the right, the average response of each material for each gas is calculated, as well as the relative selectivity. It is noted that the greatest response for all gases comes from the pr3 sensor, which is therefore the most sensitive. Furthermore, as already seen, the pr3 sensor is also the most selective, with a selectivity value of 1.63.

However, it is evident that the trend changes depending on the temperature and gas concentration. Only the pr2 sensor has a unique selectivity for acetone (all values are greater than 1), while the others have a selectivity that oscillates between acetone and ethanol. The pr3 sensor has a selectivity for acetone that decreases as the temperature increases and, at 500 °C, it becomes selective for ethanol. In the other sensors, the situation varies even at the same temperature, making the situation more complex. However, what appears confusing in Figure S3, is very useful for the realization of an electronic nose, since the different behaviors that a material has at different temperatures provide valuable information to the algorithms.

**Figure 4:**
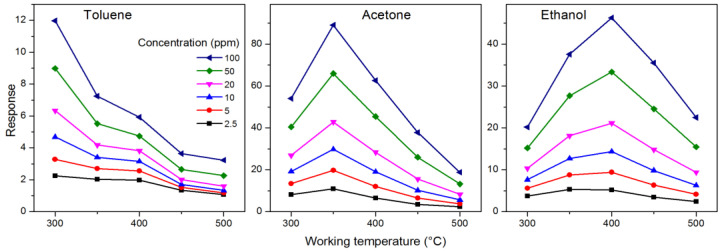
Thermal fingerprints: the response of the pr3 sensor to various gases as a function of the working temperature and concentration.

A simple method to visualize the selectivity of a sensor is to plot the thermal fingerprints related to the various gases. Figure 4 shows, as an example, the fingerprints related to the various concentrations of the three VOCs tested with the pr3 sensor. The different trend of the response as a function of temperature for the different gases is evident: the response to toluene is maximum at 300 °C, and then decreases monotonically; the response to acetone has a maximum at 250 °C; and the response to ethanol has its maximum at 400 °C. These different behaviors, or in other words, the correlations between the responses at the different temperatures, contain the information that is processed by the algorithms to distinguish the gases and subsequently estimate their concentration. In fact, the thermal fingerprints shift upwards as the gas concentration increases, but maintain the same shape, typical of that gas.

The fingerprints in Figure 4 are shown on different scales to better appreciate their trends, but in Figure S4 they are reported on the same scale, so as to be able to compare their absolute intensity. It is evident here that the response to toluene is the lowest, while that to acetone is usually the highest, even though at high temperatures it is comparable to that of ethanol. The intensity of the fingerprint response is important, but the shape of the fingerprints themselves is more important.

**Figure 5:**
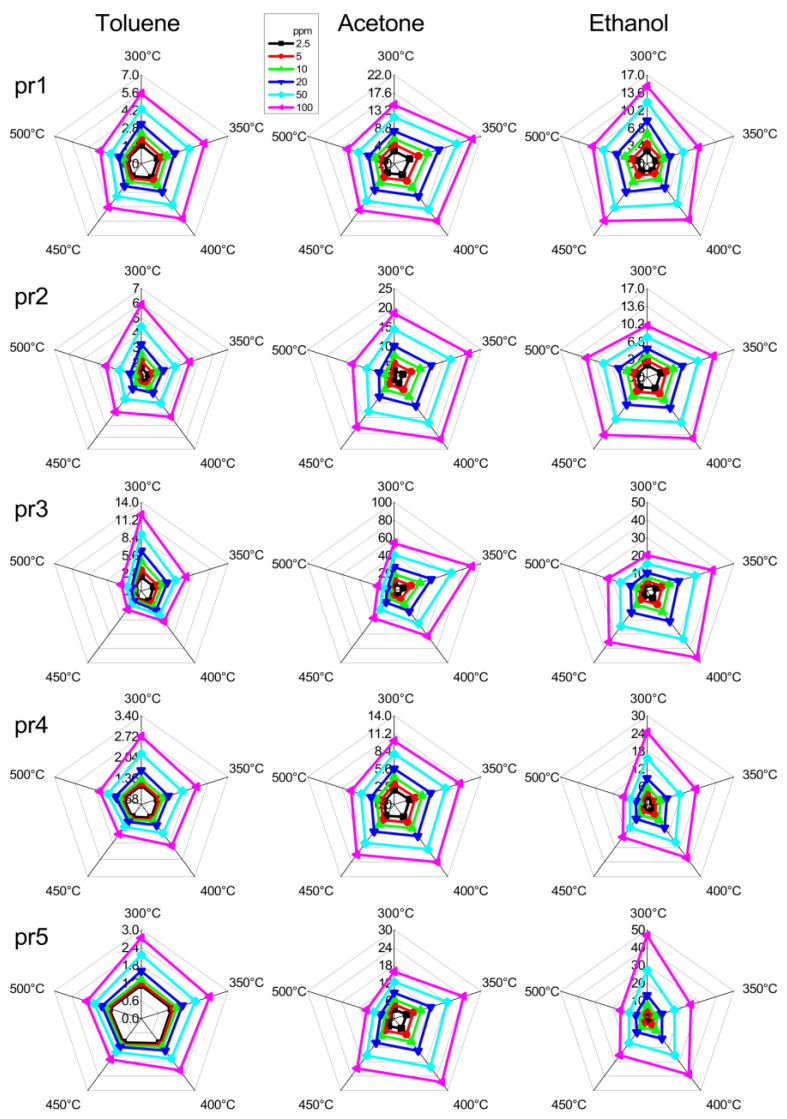
Radar plots obtained from different sensors (rows) in response to the three VOCs (columns). The five axes in each radar plot report the response values at different temperatures.

Another way to qualitatively see the selectivity of a sensor (commonly used in scientific papers) is to compare the radar plots in Figure 5. The greater the difference in the radar plots for the different VOCs are, the more we expect the sensor to be intrinsically selective. Unfortunately, this method is only effective in the simplest cases (few gases and very different fingerprints) because the human eye is not as good at this task as a mathematical algorithm. Looking at Figure 5, we can see that pr3 seems more selective than pr1 or pr2, but it is not easy to go into more detail.

**Figure 6:**
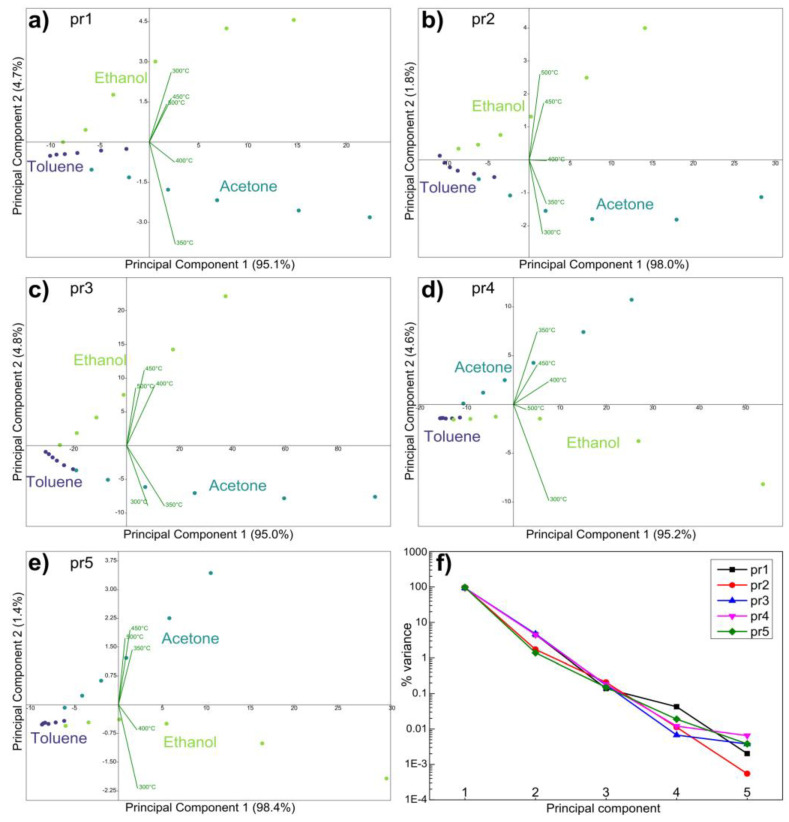
(**a**–**e**) PCA plots obtained with the different sensors; (**f**) percentage of variance explained by the principal components for each sensor.

For this reason, each thermal fingerprint in Figure 4 was transformed into a five-dimensional point and processed with classification and regression algorithms. Initially, a principal component analysis of the data was performed to reduce the dimensionality and verify how the data from the various gases are related to each other. Figure 6a–e shows plots of the first two principal components obtained with the data of the five sensors. It can be seen that, in all the panels, the points related to each gas lie on fairly straight lines. The lines always start from a common area on the left (when the concentration is low, all gases are similar to air) and then fan out in different directions at higher concentrations. It is therefore clear, in a qualitative way, that the thermal electronic nose will have fewer problems distinguishing gases at higher concentrations. Unfortunately, these very long lines make it impossible to use untrained classification algorithms. In fact, they are based on the distance between the points, and the low-concentration points of ethanol and acetone are closer to the toluene points than to the high-concentration points of acetone and ethanol, so they would be confused by this type of algorithm. However, this arrangement of the points will be useful in the next regression step.

Figure 6f shows the percentage of variance explained by the principal components for the various sensors. The sensors behave very similarly, with PC1 explaining 95–98%, PC2 demonstrating 1.4–4.8%, and the other PCs explaining less than 0.25% each. This means that the plots in Figure 6 show almost all the variance of the data in the five-dimensional space, and thus explain the relationships between the points very well. Furthermore, they indicate that the sensor responses at different temperatures are highly correlated with each other, as expected.

**Figure 7:**
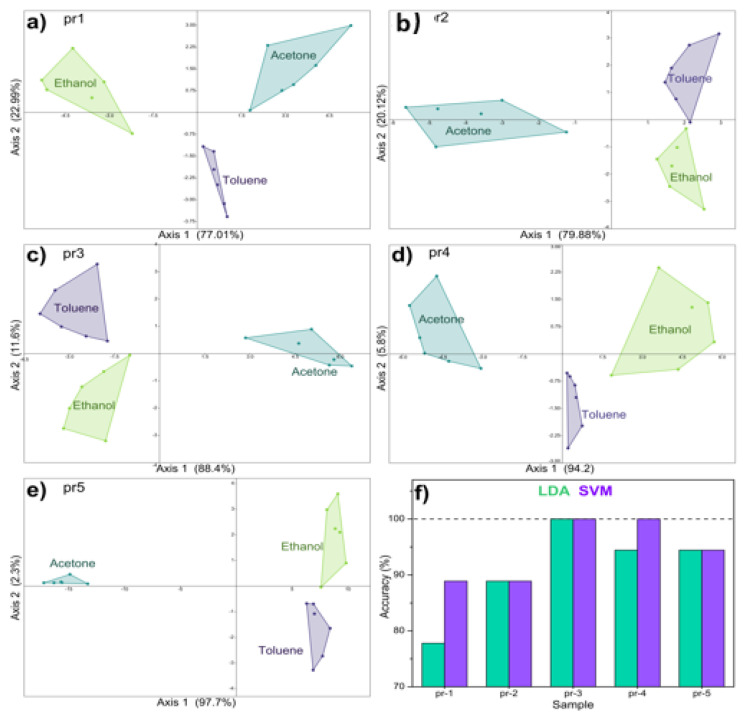
(**a**–**e**) LDA graphs obtained with the different sensors; (**f**) accuracy of each sensor calculated with LDA and SVM.

Linear discriminant analysis (LDA) was used to measure how well the thermal electronic nose can distinguish the various VOCs. The panels in Figure 7a–e show the LDA plots obtained with the responses of the five sensors. All the nanomaterials distinguish the points in three separate clusters related to the three VOCs. The quantitative classification obtained by the LDA is reported in Figure 7f. The percentage of correct classifications goes from 78% up to 100% for the pr3 sensor and then drops to 94.4%. The data were also tested with a support vector machine used as a classifier, with an accuracy of 100% for the pr3 and pr4 sensors.

**Figure 8:**
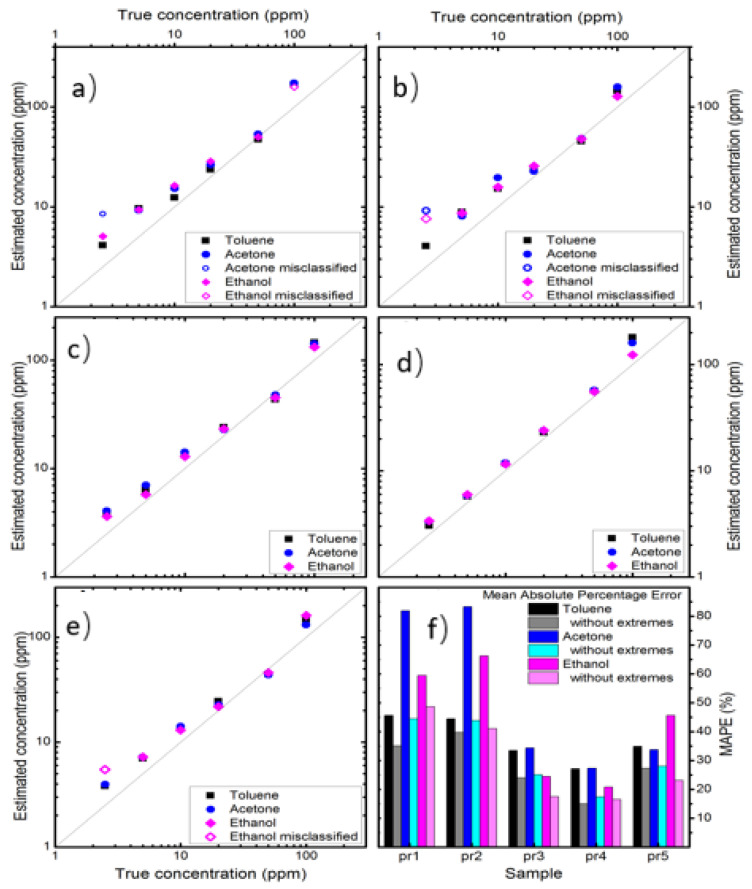
(**a**–**e**) Estimated concentration versus true concentration for the various sensors; (**f**) calculated error for the three VOCs with each sensor.

Once classified, the points were fed to three 5D SVM regressors that estimated the gas concentration. The panels in Figure 8a–e show the concentration estimated by the thermal electronic nose as a function of the actual concentration injected into the measurement chamber. The diagonals indicate the perfect estimate. Correctly classified measurements are indicated as solid points, while incorrect ones are empty circles. In general, the points are quite close to the diagonal, indicating a good accuracy of the electronic nose. At first glance, it can be noticed that the pr3 and pr4 sensors show more accurate estimates, i.e., closer to the diagonal. Points at the extremes of the measurement range tend to be further from the diagonal, i.e., to have a larger error. This is understandable, since the electronic nose is less trained at low and high concentrations. Furthermore, points at low concentrations actually seem less accurate than those at high concentrations. This is due to three contributions: (i) less training of the electronic nose, (ii) greater error in the sensor measurements since it is closer to the limit of detection, and (iii) greater similarity of the thermal fingerprints since the more diluted the gases are, the more they all resemble air. The mean absolute percentage error (MAPE) calculated for each sensor is reported in Figure 8f. To account for the lower training at the ends of the measurement range, the MAPE was also calculated excluding the lowest and the highest concentration (this is reasonable, since electronic noses are usually trained on a wider range than the one used in applications).

Figure 8f reports the error for each sensor at each gas, and it is seen that the nanocomposites with higher amounts of Au (pr3, pr4, and pr5) perform better. The average error is also calculated on the misclassified measurements, which have larger errors because a wrong regressor is used. Despite this, the pr4 sensor has an error of 27.1%, 27.3%, and 20.8% for toluene, acetone, and ethanol, respectively. It should be noted that the concentration is a quantity that spans many orders of magnitude, so an error of 20–30% is useful in many applications. Eliminating the measurements at the extremes of the measurement range, due to limited training, the error of pr4 sensor is 15.0%, 17.4%, and 16.5% for toluene, acetone, and ethanol, respectively.

In conclusion, the Cu_2_O–SnO_2_ sensor with 4 wt% of Au achieves a correct classification in 100% of the cases with the SVM and provides the best estimate of the gas concentration. This is in contrast to the measurement of the average intrinsic selectivity of the material (in Figure 3a) and demonstrates that the fabrication of a thermal electronic nose should be studied in more detail, not relying only on the intrinsic performance of the material used as a sensor. The sensor’s performance (perfect classification of acetone, ethanol, and toluene with an average concentration error of about 16%), together with its potentially micrometric dimensions, make this type of electronic nose ideal for several applications, including rapid screening for diabetes detection.

## 4. Conclusions

Different chemoresistive gas sensors based on porous Cu_2_O–SnO_2_ nanospheres have been fabricated by adding different amounts of Au (from 1 to 5 wt%). The sensors have been used to fabricate thermal electronic noses (i.e., electronic noses that use different temperatures instead of different materials) to distinguish acetone, ethanol, and toluene and estimate their concentrations. Although the sensor with the highest intrinsic selectivity is the one with 3 wt% Au, the best performance as an electronic nose is achieved by the sensor with 4 wt% Au, with a classification accuracy of 100% and an error on the concentration estimation around 16%. These performances, combined with the possibility of fabricating the device in less than one square millimeter, make the thermal electronic nose based on porous Cu_2_O–SnO_2_ nanospheres with 4 wt% Au an ideal candidate for portable, integrable, and wearable devices.

## Data Availability

The data that support the findings of this study are available from the corresponding author upon reasonable request.

## Supplementary Materials

The following supporting information can be downloaded at: https://www.mdpi.com/article/10.3390/nano14242052/s1, Figure S1: XRD spectra of the Cu-SnO_2_ powders with different amounts of Au; Figure S2: Relationship between response to acetone and response to ethanol as a function of material, temperature and gas concentration. On the right, average response to various VOCs and average selectivity; Figure S3: Thermal fingerprints obtained with pr3 sensor for the three VOCs at different concentrations. Figure S4. Thermal fingerprints obtained with pr3 sensor for the three VOCs at different concentrations
