# Supplementary material for: Effects of Au Addition on the Performance of Thermal Electronic Noses Based on Porous Cu2O–SnO2 Nanospheres
Source: Nanomaterials (Basel). 2024 Dec 22;14(24):2052. doi: 10.3390/nano14242052 (PMC11677366; doi:10.3390/nano14242052)
Supplement: Supplementary file 1 [file nanomaterials-14-02052-s001.zip › nanomaterials-3380764-supplementary.pdf]

Supplementary Material of
Matteo Tonezzer [1,*], Taro Ueda [2], Soichiro Torai [3], Koki Fujita [3], Yasuhiro Shimizu [3] and Takeo Hyodo [2,*]

1 Department of Chemical and Geological Sciences, University of Cagliari, 09042 Monserrato, Italy
2 Graduate School of Integrated Science and Technology, Nagasaki University, 1–14 Bunkyo-machi,
Nagasaki 852-8521, Japan; taroueda@nagasaki-u.ac.jp
3 Graduate School of Engineering, Nagasaki University, 1–14 Bunkyomachi, Nagasaki 852-8521, Japan;
torai-souichirou@fujielectric.com (S.T.); bb52123633@ms.nagasaki-u.ac.jp (K.F.); shimizu@nagasaki-u.ac.jp (Y.S.).
* Correspondence: matteo.tonezzer@unica.it (M.T.); hyodo@nagasaki-u.ac.jp (T.H.)

The spectra in Fig. S1 clearly show that the intensity of the Au (200) and (311) peaks increases with increasing amount of Au present.

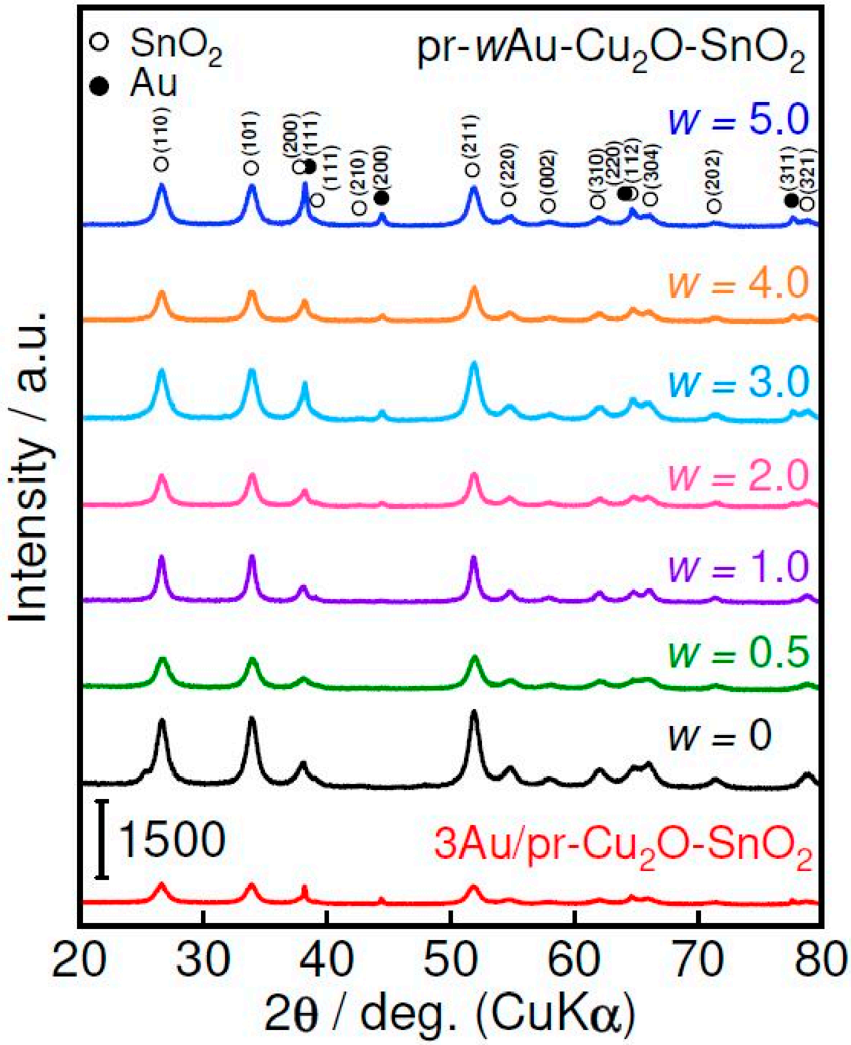

Figure S1. XRD spectra of the Cu-SnO2 powders with different amounts of Au [S1].

Academic Editor: Ioannis V. Yentekakis

Received: 4 December 2024
Revised: 17 December 2024
Accepted: 20 December 2024
Published: 22 December 2024

Citation: Tonezzer, M.; Ueda, T.; Torai, S.; Fujita, K.; Shimizu, Y.; Hyodo, T. Effects of Au Addition on the Performance of Thermal Electronic Noses Based on Porous Cu_2O–SnO_2 Nanospheres. *Nanomaterials* **2024**, *14*, 2052. https://doi.org/10.3390/nano14242052

Copyright: © 2024 by the authors. Licensee MDPI, Basel, Switzerland. This article is an open access article distributed under the terms and conditions of the Creative Commons Attribution (CC BY) license (https://creativecommons.org/licenses/by/4.0/).

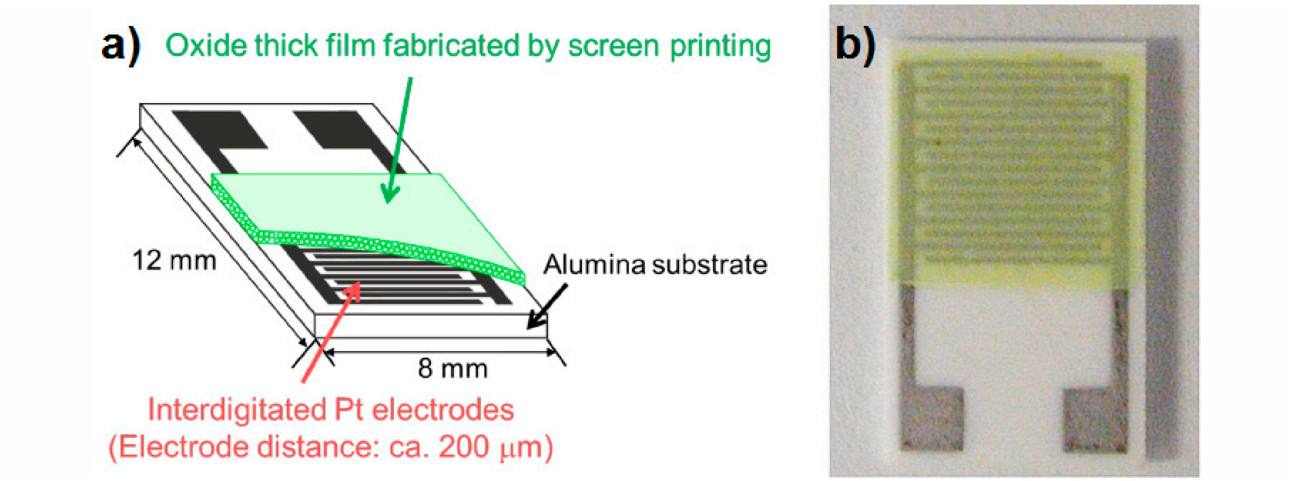

Figure S2. a) Schematic of the interdigitated sensor, b) photograph of the sensor. Modified from [25].

In the colored area, Fig. S3 reports the ratio of the response to acetone to the response to ethanol for the five sensors, as a function of temperature and gas concentration. When the value is greater than 1, it coincides with the sensor selectivity, while when it is lower, the selectivity is its reciprocal.

On the right is the average of the response to each of the three VOCs (the largest is highlighted), and further down is the average selectivity of the sensor, calculated as the ratio of the two largest responses.

The heat map gives a quick idea of how much more complicated the situation is than the selectivity calculated by averaging all the measurements, since the ratio varies not only greatly from sensor to sensor, but also as a function of temperature and even gas concentration. These differences are exploited for the operation of the thermal electronic nose.

	Concentration (ppm)	Selectivity Acetone / Ethanol ratio 300°C	350°C	400°C	450°C	500°C	Average	
pr1	2.5	1.29	2.22	2.06	1.60	1.24	2.60	Toluene
	5	1.28	2.23	2.16	1.53	1.17	8.54	Acetone
	10	1.08	2.62	2.02	1.36	1.12	6.20	Ethanol
	20	0.97	2.51	1.76	1.20	1.09		
	50	0.98	2.25	1.48	1.10	1.07	1.38	Selectivity
	100	0.99	1.96	1.32	1.05	1.11		
pr2	2.5	1.59	1.63	1.53	1.21	1.15	2.49	Toluene
	5	1.72	1.69	1.52	1.26	1.14	9.61	Acetone
	10	1.81	1.73	1.54	1.26	1.11	6.45	Ethanol
	20	1.86	1.72	1.55	1.28	1.09		
	50	1.88	1.71	1.54	1.31	1.08	1.49	Selectivity
	100	1.89	1.67	1.51	1.32	1.10		
pr3	2.5	2.19	2.06	1.25	1.00	0.97	3.61	Toluene
	5	2.39	2.25	1.28	1.02	0.92	24.88	Acetone
	10	2.51	2.35	1.33	1.03	0.90	15.25	Ethanol
	20	2.60	2.36	1.34	1.05	0.88		
	50	2.66	2.38	1.36	1.06	0.86	1.63	Selectivity
	100	2.68	2.37	1.35	1.06	0.84		
pr4	2.5	1.05	1.97	1.56	1.49	0.90	1.72	Toluene
	5	0.98	1.96	1.41	1.39	0.81	9.91	Acetone
	10	0.85	1.77	1.17	1.20	0.74	12.06	Ethanol
	20	0.67	1.48	0.97	1.05	0.72		
	50	0.46	1.17	0.79	0.91	0.67	1.22	Selectivity
	100	0.34	0.95	0.68	0.81	0.63		
pr5	2.5	0.87	1.19	0.91	1.09	0.98	1.45	Toluene
	5	0.83	1.11	0.80	1.03	0.98	5.31	Acetone
	10	0.75	1.00	0.70	0.94	0.97	7.68	Ethanol
	20	0.64	0.87	0.63	0.88	0.96		
	50	0.51	0.73	0.56	0.78	0.91	1.44	Selectivity
	100	0.41	0.63	0.50	0.72	0.85		

Figure S3. Relationship between response to acetone and response to ethanol as a function of material, temperature and gas concentration. On the right, average response to various VOCs and average selectivity.

Fig. S4 shows the thermal fingerprints reported in Fig. 4, but on the same intensity scale, so that they are more comparable. It is therefore noted that the response to toluene is always much lower than the others, while in general the response to acetone is the highest (except at high temperatures, where it is comparable with the response to ethanol).

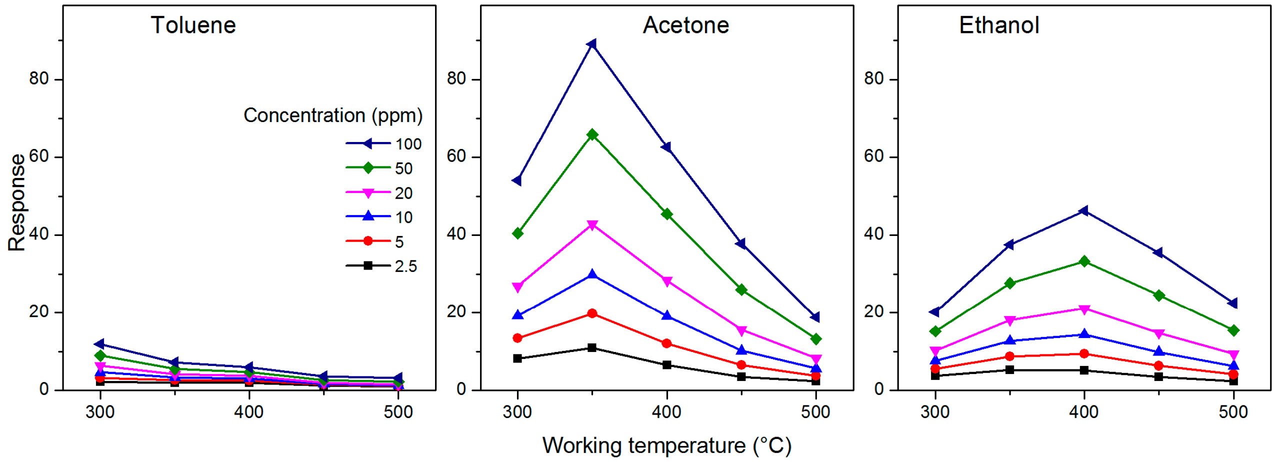

Figure S4. Thermal fingerprints obtained with pr3 sensor for the three VOCs at different concentrations.

[S1] Ueda, T.; Torai, S.; Fujita, K.; Shimizu, Y.; Hyodo, T. Effects of Au Addition to Porous CuO_2-Added SnO_2 Gas Sensors on Their VOC-Sensing Properties. *Chemosensors* **2024**, 12, 153.
